# Estimation of minute ventilation by heart rate for field exercise studies

**DOI:** 10.1038/s41598-020-58253-7

**Published:** 2020-01-29

**Authors:** Ramon Cruz, Danilo L. Alves, Eduardo Rumenig, Renata Gonçalves, Edson Degaki, Leonardo Pasqua, Sarah Koch, Adriano E. Lima-Silva, Michael S. Koehle, Romulo Bertuzzi

**Affiliations:** 1 Endurance Performance Research Group (GEDAE-USP), School of Physical Education and Sport, University of São Paulo, São Paulo, SP Brazil; 2 Physical Performance Research Group, Federal University of Parana, Curitiba, PR Brazil; 3 School of Kinesiology, University of British Columbia, Vancouver, BC Canada; 4 Human Performance Research Group, Academic Department of Physical Education, Federal University of Technology – Parana (UTFPR), Curitiba, PR Brazil; 5 Faculty of Medicine, University of British Columbia, Vancouver, BC Canada; 6 Sports Center, Federal University of Santa Catarina, Florianópolis, SC Brazil

**Keywords:** Public health, Weight management

## Abstract

The aim of this study was to develop predictive equations for minute ventilation based on heart rate, and to test the precision of the equations in two forms of endurance exercise. Eighteen men (age 27.8 ± 5.4 years old, maximal oxygen uptake 45.4 ± 8.3 ml·kg^−1^·min^−1^) performed a maximal progressive cycle test in which minute ventilation and heart rate were continually measured and further used to establish the proposed equations with quadratic and exponential adjustments. In the second and third laboratory visits, sixteen participants completed two cycling bouts, one high-intensity interval exercise and one low-intensity continuous exercise. The minute ventilation and heart rate were measured in both exercises and the validity of the equations tested. The Bland-Altman analysis showed agreement between the minute ventilation and estimated equations for interval and continuous exercise. There was no difference between the minute ventilation obtained from both equations and the minute ventilation directly measured during the interval exercise. However, the quadratic equation underestimated the minute ventilation during continuous exercise (p < 0.05). While both equations seem to be suitable to estimate minute ventilation during high-intensity interval exercise, the exponential equation is recommended for low-intensity continuous exercise.

## Introduction

In field exercise studies, researchers are sometimes unable to directly measure important variables that characterize the respiratory response to a stimulus or an intervention, such as minute ventilation ($\mathop{{\rm{V}}}\limits^{.}$E). Often, the required equipment to measure this variable is expensive, delicate to transport to remote areas, susceptible to ambient humidity and temperature fluctuations, or their maintenance is costly. Nevertheless, for some field studies investigating the effects of physical activity on health, the assessment of $\mathop{{\rm{V}}}\limits^{.}$E is imperative. For example, studies investigating the interaction between air pollution and physical activity on health in a “real-world” setting^1–9^ depend on the measurement of $\mathop{{\rm{V}}}\limits^{.}$E; however, a direct measurement of this respiratory parameter may be impossible.

It is believed that there is an exponential relationship between $\mathop{{\rm{V}}}\limits^{.}$E and exercise intensity^9^. Furthermore, some methodological issues make the direct measurement of $\mathop{{\rm{V}}}\limits^{.}$E during exercise in air pollution exposure difficult: (1) it is often not feasible to evaluate a large number of participants, such as during endurance exercise in big cities (2). Alternatively, there is the possibility that facemasks or mouthpieces used to assess exhaled air create a physical barrier to the pollutants, generating a microclimate inside the facemask or mouthpiece and thus interfering with the purposes of a study.

Due to these challenges assessing $\mathop{{\rm{V}}}\limits^{.}$E in field studies, previous research has proposed indirect forms to estimate breath-related parameters^9–11^. For example, Valli *et al*.^10^ and Onoratti *et al*.^11^ have demonstrated how the $\mathop{{\rm{V}}}\limits^{.}$E and the heart rate (HR) could be used to detect ventilatory compensation point (VCP). Another study conducted by Zuurbier *et al*.^9^ established a predictive equation for $\mathop{{\rm{V}}}\limits^{.}$E during commuting with cyclists, car drivers and bus passengers. In their study, the HR and $\mathop{{\rm{V}}}\limits^{.}$E were highly correlated (r = 0.90; p < 0.05; n = 34) and the equation was established during sub-maximal incremental cycling test (under of 80% of HR max). It is intriguing that the relatively easy and cost-efficient assessment of HR could be used to estimate $\mathop{{\rm{V}}}\limits^{.}$E in future environmental health studies. They would (1) reduce the costs of needed equipment; (2) permit the evaluation of $\mathop{{\rm{V}}}\limits^{.}$E of participants during exercise in outside environment such as parks and city streets, and (3) there would be no “physical barrier” between the pollutants and the mouth.

Considering the exponential relationship between $\mathop{{\rm{V}}}\limits^{.}$E and exercise intensity, it is plausible that during high-intensity exercise bouts, $\mathop{{\rm{V}}}\limits^{.}$E will be higher than the values observed in the sub-maximal incremental cycling test performed by Zuurbier *et al*.^9^. Thus, before establishing the equation, it is important to consider a test with exercise intensity ranging from low to high to establish an equation that covers all exercise intensities. Once established, the equation would require validation with different exercise protocols to ensure that the equation remains valid with different protocols such as high-intensity interval exercise and low-intensity continuous exercise. Therefore, the aim of the present study was to develop a predictive equation to estimate $\mathop{{\rm{V}}}\limits^{.}$E based on HR responses that would be valid across exercise intensities (*i.e*. low- and high-intensities) and modes (*i.e*. continuous and interval exercise).

## Methods

### Participants

Eighteen physically active males (age 27.8 ± 5.4 years, body mass 71.4 ± 9.1 kg, height 175.8 ± 8.7 cm, body mass index 23.0 ± 1.6 kg·m^−2^, and maximal oxygen uptake ($\mathop{{\rm{V}}}\limits^{.}$O_2_max) 45.4 ± 8.3 ml·kg^−1^·min^−1^) participated in this study. Participants were classified as recreationally trained^12^. They were excluded if they presented any cardiorespiratory disease, were on regular medications or anabolic steroids, or if they had recent injuries that compromised their participation. Those who met the eligibility criteria were recruited as a volunteer to participate, and the convenience sample was used for this study. The participants were informed about the risks associated with the study protocol and provided written, informed consent. This investigation was approved by the Ethics Committee for Human Studies of the University of Sao Paulo in accordance with international standards^13^.

### Experimental design

Each participant visited the laboratory on three occasions. In the first visit, they completed anthropometric assessments and a cycling maximal incremental test to determine their $\mathop{{\rm{V}}}\limits^{.}$O_2_max, and the response of $\mathop{{\rm{V}}}\limits^{.}$E and HR to the increment of exercise intensity. On the second and third visits, two participants did not perform the tests for personal reasons. Thus, sixteen participants performed a high-intensity interval exercise or a low-intensity continuous exercise, both on a cycle ergometer. During both protocols, HR and $\mathop{{\rm{V}}}\limits^{.}$E were measured. The participants were asked to refrain from vigorous physical activities, caffeine and alcohol 48 h before each experimental session.

### Maximal incremental test

The maximal incremental test was carried out on a mechanically braked cycle ergometer (Biotek 2100, Cefise, Nova Odessa, SP, Brazil). After a 5-minute warm-up at 30 W, work rate was increased by 30 W every minute until volitional fatigue. Participants cycled at a cadence of 60–70 revolutions per minute (rpm) and volitional fatigue was defined when participants were unable to maintain cadence above 60 rpm. Participants received strong verbal encouragement to continue the test as long as possible. Our participants reached volitional fatigue in 08:51 ± 01:35 minutes. Gas exchange was measured breath-by-breath with a metabolic cart (Cortex Metalyzer 3B, Cortex Biophysik, Leipzig, Germany), and 30-second means were subsequently calculated for further analysis of $\mathop{{\rm{V}}}\limits^{.}$E. $\mathop{{\rm{V}}}\limits^{.}$O_2_max was determined when at least two of the following criteria were met: an increase in oxygen uptake of less than 2.1 ml·kg^−1^·min^−1^ between two consecutive stages, a respiratory exchange ratio greater than 1.1 and the attainment of a HR greater than 90% of the predicted maximal HR (i.e., 220-age)^14^. HR was monitored during the test by a HR monitor (model S810; Polar Electro Oy, Kempele, Finland).

### Endurance cycling protocols

The two endurance cycle protocols were carried out on the same cycle ergometer used for the maximal exercise test. After a 5-min warm-up at 30 W, participants performed a high-intensity interval exercise composed of 10 repetitions of one minute at an intensity corresponding to 100% of $\mathop{{\rm{V}}}\limits^{.}$O_2_max (work rate where 100% $\mathop{{\rm{V}}}\limits^{.}$O_2_max occurred), followed by one minute at an intensity corresponding to 40% of $\mathop{{\rm{V}}}\limits^{.}$O_2_max (work rate where 40% $\mathop{{\rm{V}}}\limits^{.}$O_2_max occurred). This exercise protocol has been often indicated for developing the cardiorespiratory health^15^. The total work accomplished during high-intensity interval exercise was calculated and the duration of the low-intensity continuous exercise was fixed to match with the work performed during the high-intensity interval. Thus, the low-intensity continuous exercise was performed at 40% of $\mathop{{\rm{V}}}\limits^{.}$O_2_max during 38.2 ± 2.9 minutes. Participants exercised at a pedal cadence of 60–70 rpm in all tests. The $\mathop{{\rm{V}}}\limits^{.}$E was measured using the same metabolic cart described above, as well the HR during the endurance cycling protocols.

### Estimation of minute ventilation and validation of the equation

First, the $\mathop{{\rm{V}}}\limits^{.}$E was plotted against the HR. Then, the curve estimation test was performed to select the best adjustment between the variables (measured by R square – R²). In this sense, only equations with R² superior of 0.90 were selected. The equations were individually established based on the relationship between $\mathop{{\rm{V}}}\limits^{.}$E and HR during the maximal incremental test, after we present the mean of each constant^9^. The $\mathop{{\rm{V}}}\limits^{.}$E was inserted into the model as a dependent variable and the HR as an independent variable. The variables were adjusted using a quadratic (*F*(*x*) = (*a* + *b*1*x*^2^ + *b*2*x*^2^)) and an exponential model (*F*(*x*) = exp(*a* + *b*1*x*)), as previously performed^9^. In the equations, the constant “a” represents the intercept, “b_1_” and “b_2_” the constants. In addition, the R² values from the equations were R² = 0.95 (p < 0.05, n = 18) and R² = 0.94 (p < 0.05, n = 18) to quadratic and exponential adjustments, respectively. After, to test if the equations could be used in non-incremental exercises, they were applied to estimate the $\mathop{{\rm{V}}}\limits^{.}$E during high- and low-intensity exercises.

### Statistical analysis

Data are presented as means and standard deviations (SD). The coefficient of determination (R²) for each equation was calculated to establish the association between $\mathop{{\rm{V}}}\limits^{.}$E and HR. Intra-class correlation (ICC) was performed to assess the absolute agreement between the measured $\mathop{{\rm{V}}}\limits^{.}$E and estimated $\mathop{{\rm{V}}}\limits^{.}$E from equations, the mean of the ICC values was present. To compare the results obtained from the two predictive equations and $\mathop{{\rm{V}}}\limits^{.}$E measured the Bland–Altman plots^16,17^ and the analysis of variance (ANOVA) of repeated measures with Tukey’s *post hoc* test were performed. To estimate the total volume inhaled, the mean of $\mathop{{\rm{V}}}\limits^{.}$E was multiple by exercise duration. Total volume of air inhaled in litters (L) was analyzed by two-way ANOVA with repeated measures considering exercise (interval and continuous) *vs* method (direct measurements and predictive equations). The Tukey’s *post hoc* test was performed to identify the differences when necessary. The Eta-square (η2) was reported for all ANOVA analysis. Statistical significance was set at p < 0.05.

## Results

Table 1 presents estimative coefficients of both equations. The average R² was 0.92 (±0.02, all p < 0.01). Both equations showed high values of ICC.

**Table 1:** Coefficients and ICC for quadratic and exponential adjustment of $\mathop{{\rm{V}}}\limits^{.}$E and HR relationship during the maximal incremental test (n = 18).

Equation	Intercept	b1	b2	ICC
Quadratic	141.324 (73.126)	−2.561 (1.144)	0.014 (0.044)	0.95 (0.06)**
Exponential	1.162 (0.475)	0.021 (0.003)	—	0.96 (0.05)**

Data are presented as means (SD). b1. Constant 1 of the equation. b2. Constant 2 of the equation. ICC. Intra-class correlation (**p < 0.01).

Data are presented as means (SD). b1. Constant 1 of the equation. b2. Constant 2 of the equation. ICC. Intra-class correlation (**p < 0.01).

**Figure 1:**
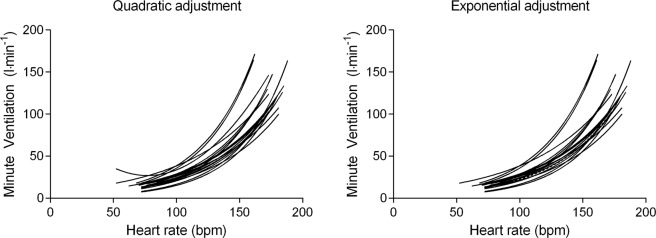
Curve estimation based on minute ventilation (V̇E) and heart rate (HR) relationship during a maximal incremental test (n = 18).

Figure 1A show the relationship between $\mathop{{\rm{V}}}\limits^{.}$E and HR during maximal incremental test for each participant based on quadratic and exponential adjustments, respectively.

**Figure 2:**
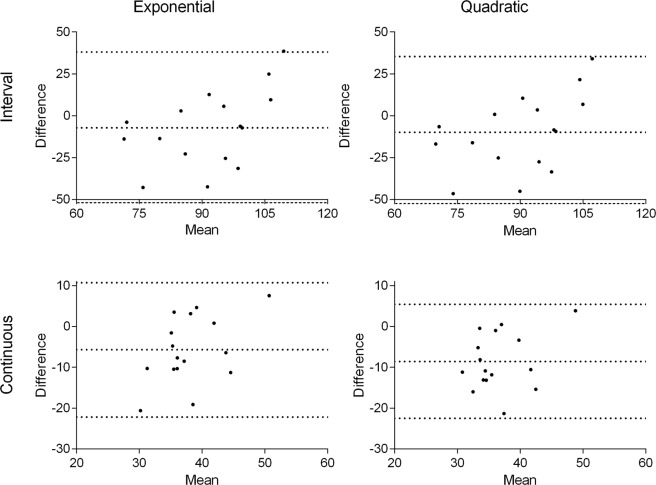
Bland Altman plots of exponential and quadratic equations for interval and continuous exercises. The dotted lines represent upper limit, mean and lower limit.

The Bland Altman analysis showed agreement between the $\mathop{{\rm{V}}}\limits^{.}$E measured and estimated by both equations for interval and continuous exercises (Fig. 2).

**Figure 3:**
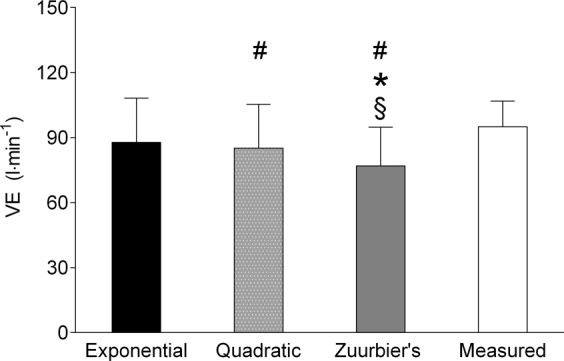
Minute ventilation (V̇E) by estimate and direct measurement during high-intensity interval exercise. Difference from direct measure: *p < 0.05. Difference from exponential equation: ^#^p < 0.05. Difference from quadratic equation: ^§^p < 0.05.

Figure 3 shows the $\mathop{{\rm{V}}}\limits^{.}$E calculated using the equations and the measured $\mathop{{\rm{V}}}\limits^{.}$E during high-intensity interval exercise. The repeated measures ANOVA indicated that there was a significant difference between the equations (F_(1, 15)_: 572.39; p < 0.001; η²: 0.97). Wherein, there was no difference between the exponential and quadratic equations adjustments and the direct measurements. Nevertheless, there was difference between Zuurbier’s equation and direct measurements.

**Figure 4:**
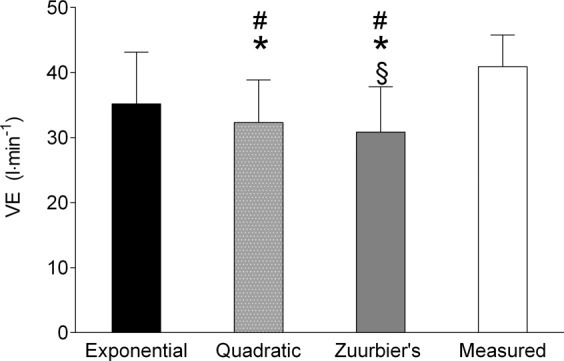
Minute ventilation (V̇E) by estimate and direct measurement during low-intensity continuous exercise. Difference from direct measure: *p < 0.05. Difference from exponential equation: ^#^p < 0.05. Difference from quadratic equation: ^§^p < 0.05.

For the low-intensity continuous exercise (Fig. 4), the repeated measures ANOVA indicated there was a significant difference between the equations and directly measured ventilation (F_(1, 15)_: 633.76; p < 0.001; η²: 0.98). There were no differences between the exponential equations and the direct measure of $\mathop{{\rm{V}}}\limits^{.}$E. There was, however, a difference between the quadratic equation and the direct measure of $\mathop{{\rm{V}}}\limits^{.}$E. In addition, there was difference between Zuurbier’s equation and direct measure.

**Figure 5:**
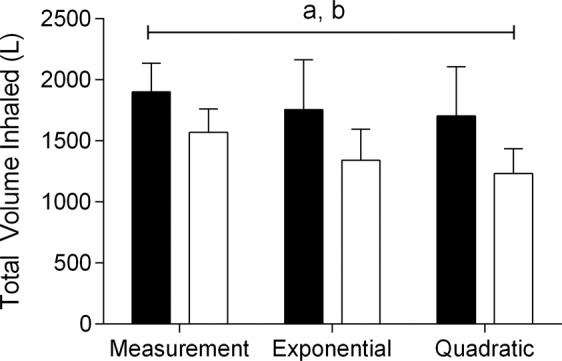
Total volume inhaled during a high-intensity interval exercise or a low-intensity continuous exercise. (**a**) Main effect of exercise. (**b**) Main effect of method to evaluate the total volume inhaled. ■ High-intensity interval exercise. □ Low-intensity continuous exercise.

Figure 5 shows the total volume measured and estimated during interval and continuous exercises (mean of $\mathop{{\rm{V}}}\limits^{.}$E multiplying by exercise duration). There was no significant interaction between the exercises and method to evaluate the total volume inhaled (F_(2, 30)_ = 1.97, p = 0.15, η^2^ = 0.12). However, there was a main effect for exercise (F_(1, 15)_ = 32.27, p < 0.001, η^2^ = 0.68). The results from *post hoc* analysis showed higher values of total volume inhaled for interval than for continuous exercise. There was also a main effect for method (F_(2, 30)_ = 7.61, p = 0.002, η^2^ = 0.34), where in the *post hoc* analysis showed lower values of total volume inhaled for exponential and quadratic equations than for direct measurement.

## Discussion

The results obtained from this study show that the two generated and validated equations can predict $\mathop{{\rm{V}}}\limits^{.}$E from HR rate with either exponential (*VE* = exp(1.16 + 0.021 × *HR*) or quadratic (*VE* = 141.324 − 2.56 × *HR* + 0.014 × *HR*^2^) adjustments. Considering the Bland Altman plots and the validation tests, it was indicated that for high-intensity interval exercise, both equations could be used, but exponential adjustment is preferable as the estimated values were closer to the direct measured $\mathop{{\rm{V}}}\limits^{.}$E. In contrast, for low-intensity continuous exercise, only the exponential equation should be used. The interval exercise resulted in a greater total volume of inhaled air compared to continuous exercise, regardless the method used to evaluate it. This is particularly important because beyond the rate of $\mathop{{\rm{V}}}\limits^{.}$E, epidemiological studies concerning environmental adverse conditions, such as air pollution, often evaluate the total volume inhaled to establish exposure-response^6,8,9,18–20^.

The high values for R² (0.92 ± 0.02; n = 18; p < 0.01) indicate that HR is a useful tool to predict the $\mathop{{\rm{V}}}\limits^{.}$E. Close R2 values for the $\mathop{{\rm{V}}}\limits^{.}$E-HR relationship have been previously described (0.90; ±0.07; n = 24)^9^. The values obtained for the intercept and the constant for the exponential adjustment previously described by Zuurbier *et al*.^9^ (1.03 ± 0.63 and 0.021 ± 0.005, respectively) (Zurbier’s equation: VE = EXP(1.03 + 0.02 ϶ HR) were very close to those observed in our study (1.16 ± 0.475; 0.021 ± 0.003, respectively). We believe our equation might be more precise because we considered more points across a large spectrum of exercise intensities (i.*e*. maximal intensity test) to establish the equation^9^. Furthermore, a recent study has indicated non-linear adjustment should also be considered for high-intensity interval exercises^18^. Therefore, in the present study we have considered two non-linear adjustments and tested their practical application in two exercises with high- and low-intensities. Interestingly, changes in the slope of increment in $\mathop{{\rm{V}}}\limits^{.}$E over HR has been applied to estimate the VCP, an important parameter for endurance exercise prescription, during an incremental test^10,11^. Furthermore, even under hypoxic conditions, the ventilatory compensation point can be reliably detected from the changes in the slope of the $\mathop{{\rm{V}}}\limits^{.}$E to HR relationship. Taking together, these studies support the application of HR to estimate alterations in breath-related parameters.

In the present study, the $\mathop{{\rm{V}}}\limits^{.}$E observed during both endurance exercises were considerably greater than $\mathop{{\rm{V}}}\limits^{.}$E observed in a previous study during commuting^9^. Comparing bus and car passengers with cyclists, Zuurbier *et al*.^9^ have demonstrated the $\mathop{{\rm{V}}}\limits^{.}$E was 2.1 times greater in cyclists; however, an equation applied to estimate the $\mathop{{\rm{V}}}\limits^{.}$E during commuting might not predict the $\mathop{{\rm{V}}}\limits^{.}$E precisely in another activities/exercise intensities. In fact, Zuurbier’s equation significantly underestimated $\mathop{{\rm{V}}}\limits^{.}$E in both exercise modes. In our study, the $\mathop{{\rm{V}}}\limits^{.}$E was approximately 2.0 and 4.3 times greater in continuous and interval exercises, respectively, when compared to cyclists from Zuurbier *et al*.^9^. These results underline that it is important to consider the intensity of the exercise when creating equations to estimate the $\mathop{{\rm{V}}}\limits^{.}$E.

A precise $\mathop{{\rm{V}}}\limits^{.}$E estimate based on HR may be important for studies concerning air pollution and endurance exercise^8,9,18^. In the setting of air pollution and respiratory health research, there are several additional issues regarding the relationship between exercise intensity and $\mathop{{\rm{V}}}\limits^{.}$E that need to be considered. Mainly because the inhaled air pollutants are affected by the breathing pattern^19^. As exercise intensity increases, ventilation increases, which leads to a higher dose of pollutants entering the airways and lungs. Furthermore, there is a predominately nasal breathing transition to oral breathing, which removes a level of filtration, potentially increasing the dose of inhaled pollutants even further^20,21^. Another factor is the depth and frequency of inhalation^9,22^, which cause a rise in pulmonary ventilation^22^. Collectively, these physiological adaptations during exercise lead to greater air pollution exposure^22,23^.

The total volume inhaled also is an important variable to air pollution studies. Our results have indicated the total volume inhaled during interval exercise was higher than continuous, regardless of the method used to evaluate this variable (directly measure or estimative equations). However, regardless of the exercise modality, the equations underestimate the total inhaled volume. In this sense using the equations over time to estimate the total volume inhaled could be inaccurate. Taking these results together, the equations can be used in comparison between exercises, but do not accurately estimate the total inhaled volume over time.

The total inhaled volume was obtained multiplying the mean of $\mathop{{\rm{V}}}\limits^{.}$E by exercise duration and even with the equalized total work, with interval exercise there were higher values of total inhaled volume than for continuous. The physiological response during exercise can support this result. During low-intensity and constant-load exercise, the pulmonary gas exchange is matched to metabolic activity, tending to the steady-state. On the other hand, during high-intensity exercise there is higher metabolic demand, thus there is a gradual increase of $\mathop{{\rm{V}}}\limits^{.}$E. Importantly, during the low-intensity periods of the interval exercise the $\mathop{{\rm{V}}}\limits^{.}$E still  over the course of the bout and continues to increase despite the low-intensity periods^24^.

Some limitations of our study must be addressed. We only considered young and non-sedentary men when establishing the $\mathop{{\rm{V}}}\limits^{.}$E equations. A previous study indicated that there are differences in the respiratory response to exercise between men and women^9^; also there are aging and physical fitness effects which could influence ventilation during exercise^25,26^. In addition, we use cycling exercise to establish the equation, in this sense its use with other populations and other activities should be considered with caution. A difference was observed between measured and estimated $\mathop{{\rm{V}}}\limits^{.}$E. However, it should be highlighted that our equations generated lower differences from measured values than a previously proposed equation^9^. Based on a simple and low-cost measure (HR), field studies, for example, those investigating air pollution and exercise could use the equations derived in the present study. Our findings may be useful to exercise physiologists for studies that require evaluation of the respiratory response to a stimulus or an intervention.

## Conclusions

In summary, we have developed and validated two equations (i.e., quadratic and exponential) to estimate $\mathop{{\rm{V}}}\limits^{.}$E from HR for two different exercise protocols, a high-intensity interval exercise and a low-intensity continuous exercise. We recommend the exponential equations for both endurance exercises.

## Data Availability

Data sharing is not applicable to this article as no datasets were generated or analysed during the current study.
